# The paradox of miR-107 in oncology: dual roles as a tumor suppressor and oncomiR

**DOI:** 10.1038/s41417-026-01042-7

**Published:** 2026-06-02

**Authors:** Mamdouh A. Ragab, Nevine Fathy, Yasmin M. Attia, Eman A. Amer, Sally A. Fahim

**Affiliations:** 1 Biochemistry Department, Faculty of Pharmacy, Ahram Canadian University, Giza, Egypt; 2 Biochemistry Department, Faculty of Pharmacy, Cairo University, Cairo, Egypt; 3 Pharmacology Unit, Cancer Biology Department, National Cancer Institute, Cairo University, Cairo, Egypt; 4 Department of Biochemistry, School of Pharmacy, Newgiza University (NGU), Giza, Egypt

**Keywords:** Cancer genetics, Cancer therapy

## Abstract

MicroRNAs (miRNAs) are central post-transcriptional regulators that shape oncogenic and tumor-suppressive networks through RNA-induced silencing complex (RISC)–mediated repression of target mRNAs. Among these, hsa-miR-107—a member of the miR-16 family encoded within an intron of the *PANK1* gene—has emerged as a particularly paradoxical regulator in cancer. Accumulating evidence indicates that miR-107 cannot be classified as a single-function miRNA; instead, it behaves as a context-dependent “molecular switch” whose impact is dictated by tissue-specific target availability, tumor stage, and the surrounding regulatory landscape. This review synthesizes mechanistic and translational data explaining how miR-107 exerts opposing roles as both a tumor suppressor and an oncomiR. In tumor-suppressive contexts, miR-107 integrates into stress-response circuitry via a p53/*PANK1*/miR-107 axis, repressing HIF-1β, impairing HIF-1 complex formation, and reducing VEGF-driven angiogenesis. miR-107 also restricts proliferation and invasion by targeting cell-cycle kinases (e.g., *CDK6* and *CDK8*), suppressing NOTCH2 signaling in glioma, and attenuating pro-survival PI3K/AKT signaling indirectly in NSCLC through *BDNF* repression. Conversely, in aggressive cancers, miR-107 promotes malignancy through “meta-oncogenic” disruption of global miRNA biogenesis by targeting *DICER1*, enabling EMT programs via loss of miR-200 family activity. It also activates PI3K/AKT signaling through direct repression of *PTEN* (notably in bladder cancer) and enhances metastatic behavior in colorectal cancer by co-silencing metastasis suppressors such as DAPK and KLF4. A distinctive layer of complexity is added by miR-107-mediated miRNA–miRNA interaction, exemplified by direct destabilization of the tumor-suppressive let-7 family in advanced breast cancer. Therefore, the review highlights higher-order regulation by lncRNA competing endogenous RNA (ceRNA) “sponges” that sequester miR-107 (e.g., LINC00662, UASR1, H19, MFI2-AS1), thereby rewiring downstream oncogenic pathways. We discuss emerging clinical implications of miR-107 as a biomarker and as a therapeutic lever for reversing multidrug resistance, while outlining key challenges for safe, context-aware miRNA-based interventions.

## Introduction: the expanding universe of microRNA regulation in oncology

### The significance of post-transcriptional regulation

MicroRNAs (miRNAs) are a group of small, evolutionarily conserved non-coding RNA molecules, which are on average 20–24 nucleotides long and have become critical regulators of gene expression [1]. They work at the post-transcriptional level and are mainly involved in regulating the stability and/or translation of target messenger RNAs (mRNAs). MiRNAs recruit the RNA-induced silencing complex (RISC) to mediate the degradation of target mRNAs or translational inhibition by binding to complementary sequences, usually in the 3′-untranslated region (UTR) of the target mRNAs [2].

Although classical models of microRNA action have focused on binding it to 3′ untranslated region (3′UTR), research has shown miRNAs have binding to 5′ untranslated region (5′UTR) and coding sequence (CDS) as well. The interactions in the 5′UTR also generally suppress translation by preventing ribosomal recruitment, but in particular cellular contexts, they may have a paradoxical activatory effect on translation [3].

On the contrary, miRNA binding in the CDS quickly pauses the process of translation elongation and often collaborates with 3′UTR sites on the same transcript to achieve optimal target repression [4]. The additional 5′UTR and CDS interactions to the target repertoire offer a broader definition of the regulatory capability of miRNAs like miR-107.

Ever since the first miRNAs were discovered, they have been known to be pleiotropic, with an immense number of fundamental biological processes being affected by them, such as cellular development, differentiation, proliferation, and apoptosis [5, 6]. The abnormality in the regulation of these powerful molecules is therefore a cornerstone in the pathology of many human diseases, with cancer being the most notable. The discovery of the regulatory layer and its processes was so important that the discovery and the mechanics of post-transcriptional gene regulation through the miRNAs pathway were awarded the 2024 Nobel Prize [1]. Within the oncology context, miRNAs may act as oncogenes (oncomiRs) or tumor suppressors, depending on the cellular context and the targets that they regulate [2].

### Canonical miRNA biogenesis as a regulatory hub

The miRNA life cycle is a highly regulated multi-phase process that, in turn, is a crucial regulatory center [1]. The recognized pathway starts in the nucleus, where miRNA genes are transcribed, usually as a part of a host gene, by RNA polymerase II to form a long primary transcript (pri-miRNA). The pri-miRNA is then cleaved by the microprocessor complex, which contains the Drosha ribonuclease III enzyme, to produce an approximately 70-nucleotide stem-loop precursor (pre-miRNA) [2]. The pre-miRNA is processed in its final step after being exported to the cytoplasm. A second RNase III enzyme, the Dicer ribonuclease, cuts the pre-miRNA stem-loop to create a short and double-stranded miRNA: miRNA* duplex. The full-grown strand of miRNA is then inserted into the RISC, where it is activated and acquires its gene-silencing activity [2]. The elements of this biogenesis pathway, and especially Dicer, are also regulated and disrupted in cancer.

### Non-canonical miRNA biogenesis pathways

Most microRNAs develop through the standard Drosha/Dicer-dependent pathway, but non-canonical biogenesis pathways introduce extra complexity to post-transcriptional regulation by avoiding one of these fundamental ribonucleases. The mirtron pathway is the most described Drosha-independent route, in which short intronic sequences are processed using pre-mRNA splicing to create pre-miRNA hairpins, which then totally bypasses the nuclear Microprocessor complex and is then exported and processed by Dicer [7].

On the other hand, Dicer-independent pathways, including miR-451 biogenesis, depend on the catalytic slicer activity of Argonaute 2 (Ago2) instead of Dicer to mature. Also, unusual miRNAs referred to as simtrons did not use either DGCR8 or Dicer, but only Drosha to form functional regulatory RNAs [8].

The identification of these varied non-canonical processes is also important in understanding intricate post-transcriptional networks, which are used in human malignancies.

### Introduction to hsa-miR-107

A particular and peculiar member of the miRNA-16 family is hsa-miR-107 (Gene ID: 406901) [9]. miR-107 is transcribed at a genomic location of specific interest: it is in an intron of the pantothenate kinase 1 (*PANK1*) gene [10]. This genomic structure is mechanically connected to the expression of its host gene, a fact that is important in the interpretation of its role in the p53 tumor suppressor pathway.

The miR-107 is a key component of the control of a myriad of oncogenic pathways. Its role is, however, very paradoxical. A thorough review of the literature shows that miR-107 may have strong carcinogenicity or, in turn, strong cancer-suppressor properties [11]. This is not an inherent role but a very contingent one, which occurs in a tissue- and cell-specific manner [2]. This review aims to clarify this apparent paradox by integrating the complex and sometimes opposing mechanisms through which miR-107 functions as a master regulator.

We are mainly concerned with the fully developed hsa-miR-107-3p strand, which is the most common and the most well-investigated guide strand in the discussed oncological settings.

## The central paradox: miR-107 as a context-dependent master regulator

### A high-level overview of contradictory roles

miR-107 is a highly context-dependent switch, and its cellular output, whether it is the promotion or inhibition of malignancy (Table 1), depends on the particular signaling landscape, the presence or absence of the target transcriptome of the tissue, and the progression stage of the tumor [2]. This renders miR-107 an ideal example of the complexity of non-coding RNA networks.

**Table 1:** The dual role of hsa-miR-107 in human malignancies.

	Cancer type	Observed role	Key validated target(s)	Primary consequence	Reference
Tumor suppressor contexts	Glioma/Glioblastoma	Tumor suppressor	*CDK6*, NOTCH2, SALL4	G1 arrest, reduced invasion	[22]
	Gastric cancer	Tumor suppressor	*CDK6*	G1 cell cycle arrest	[19]
	NSCLC	Tumor suppressor	*BDNF*, TRIAP1	PI3K/AKT inhibition, stimulate apoptosis	[12]
	Breast cancer (progression)	Tumor suppressor	*CDK8*	G0/G1 arrest, inhibit migration	[16]
	Hepatocellular carcinoma (HCC)	Suppressor/Chemosensitizer	Cofilin-1	Promote cell death, increase ROS, increase chemosensitivity	[25]
	Colorectal cancer (progression)	Tumor suppressor	TFR1	Inhibit proliferation, inhibit metastasis	[15]
OncomiR (oncogene) contexts	Bladder cancer	OncomiR	*PTEN*	PI3K/AKT activation	[13]
	Colorectal cancer (metastasis)	OncomiR	DAPK, KLF4, Par4	EMT, promote invasion, promote proliferation	[14]
	Breast cancer (advanced)	OncomiR	*let-7 (miRNA)*	Destabilizes let-7, promote malignancy	[17]
	Gastric cancer (metastasis)	OncomiR	*DICER1*	Downregulate global miRNA, promote EMT, and invasion	[26]

The best example is the master survival pathway, PI3K/AKT regulation. miR-107 is a tumor suppressor in non-small cell lung cancer (NSCLC), which suppresses the PI3K/AKT pathway indirectly [12]. Conversely, miR-107 is a definite oncogene in bladder cancer since it directly promotes the same PI3K/AKT pathway [13].

This contradiction is also observed in colorectal cancer (CRC), where the literature is directly opposed. A number of studies refer to miR-107 as an overexpressed metastatic driver (metastamiR) that facilitates invasion by acting on suppressors [14]. Other reports, nevertheless, indicate that miR-107 is a downregulated tumor suppressor in CRC, and its reintroduction suppresses metastasis [15]. This paradox can also be seen in breast cancer, in which miR-107 has been characterized as a suppressor that halts the cell cycle [16], and an oncomiR that facilitates metastasis in the late-stage disease [17].

Such contradictions cannot be considered methodological failures; they are the manifestation of a complicated biological reality in which the role of miR-107 is an emergent property of its environment.

## miR-107 as a tumor suppressor: mechanisms of growth inhibition

**Fig. 1:**
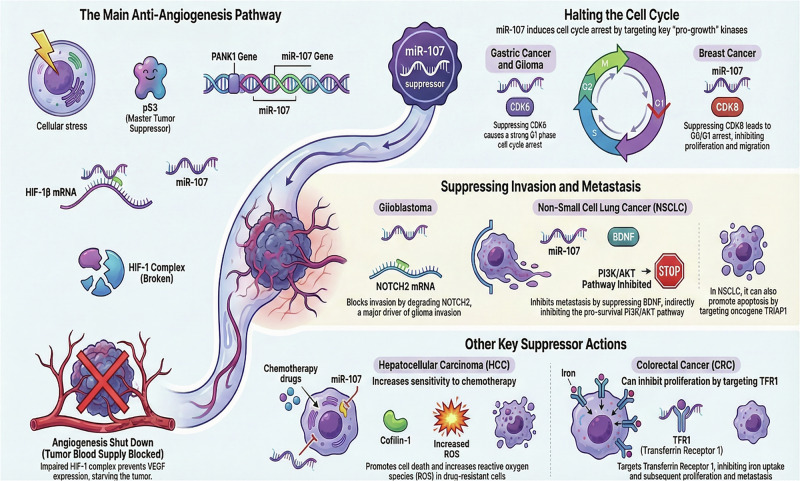
The “host-gene” tumor suppressor axis. miR-107 acts as a stress-responsive tumor suppressor through context-dependent mechanisms. p53-induced miR-107 inhibits angiogenesis by repressing HIF-1β and VEGF signaling, enforces cell-cycle arrest via targeting CDK8, and suppresses invasion and metastasis through inhibition of NOTCH2 and the BDNF/PI3K/AKT pathway. miR-107 also enhances chemosensitivity and oxidative stress in hepatocellular carcinoma by targeting cofilin-1 and inhibits proliferation in colorectal cancer via repression of transferrin.

miR-107 also acts as a powerful tumor suppressor in many cancer settings and has been shown to be downregulated in tumors. Its re-expression can stop proliferation, trigger cell cycle arrest, and suppress angiogenesis, and this is mostly achieved by acting on major oncogenic proteins, as shown in Fig. 1.

### The canonical suppressor pathway: the p53/miR-107/HIF-1 beta axis and anti-angiogenesis

The most specific tumor suppressor activity of miR-107 is the direct incorporation into the p53 pathway as an anti-angiogenic mediator [18]. This function is activated in response to cellular stress and represents a key pillar of its tumor-suppressor identity. The p53 tumor suppressor is activated in response to DNA damage (e.g., by genotoxic agents such as etoposide). Activated p53 is a transcription factor, and it has been established that it directly controls the expression of miR-107. It has been confirmed by chromatin immunoprecipitation (ChIP) experiments that p53 directly interacts with a particular p53 binding site in the promoter of the *PANK1* gene. Since the miR-107 gene is in an intron of *PANK1*, transcription of the host gene under the control of p53 also leads to miR-107 being co-transcribed [10].

This miR-107, which is induced by p53, then plays its anti-tumor role. The main one in this scenario is HIF-1 beta (Hypoxia Inducible Factor-1 beta). HIF-1 transcription factor, a heterodimer of unstable HIF-1 alpha subunit and stable HIF-1 beta subunit, is the overall controller of cellular response to hypoxia. The effect of miR-107 on HIF-1 beta mRNA is to repress the 3′-UTR of HIF-1 beta mRNA, thereby effectively impairing the cell to form the functional HIF-1 complex [10].

The functional implication is far-reaching. In the absence of the HIF-1 complex, the cell is unable to instigate a transcriptional reaction to hypoxia, which incorporates the expression of vascular endothelial growth factor (VEGF). This p53-miR-107-HIF-1 beta pathway thus reduces the expression of VEGF, prevents the differentiation of endothelial progenitor cells and tumor vascularization (angiogenesis), and ultimately the growth of tumors [10] as shown in Fig. 1.

The PANK1 gene contains the sequence for miR-107 within its intronic region. Upon activation by the p53 transcription factor in response to stress, both the host gene (PANK1) and the intronic miRNA (miR-107) are co-transcribed. During splicing, the miR-107 precursor is excised from the intron and processed. In the cytoplasm, mature miR-107 targets the 3′UTR of HIF-1 beta mRNA, preventing the formation of the functional HIF-1 alpha/HIF-1 beta complex. This results in the suppression of VEGF expression and the subsequent inhibition of tumor angiogenesis.

### Induction of cell cycle arrest: targeting cyclin-dependent kinases

The second significant repressive role of miR-107 is the fact that it silences the cell cycle by directly silencing the kinases, which promote proliferation. miR-107 is a proven tumor suppressor in gastric cancer. It was observed that its ectopic expression is a direct target and suppresses the mRNA and protein of Cyclin-Dependent Kinase 6 (*CDK6*). One of the prominent kinases is *CDK6*, which facilitates the transition of G1 to S. miR-107 inhibits gastric cancer cell proliferation and invasion by inducing a strong G1 cell cycle arrest by suppressing *CDK6* [19]. This mechanism is also observed in glioma, where p53-induced miR-107 has been shown to downregulate *CDK6* [20].

Similarly, in breast cancer cell lines (e.g., MDA-MB-231), miR-107 acts as a suppressor by directly targeting the 3′-UTR of *CDK8*. Overexpression of miR-107 in these cells leads to G0/G1 arrest and a significant inhibition of cell proliferation and migration [16].

### Suppressing invasion via suppressing the NOTCH2 signaling pathway

The miR-107 was observed to be downregulated in glioblastoma (GBM), which has the worst primary brain tumor mortality [21]. Its reinstatement is potently tumor-suppressive in that it targets a variety of mediators of glioma pathogenesis.

The most noticeable one in this respect is NOTCH2. Notch signaling pathway is a major proliferation and invasion driver of glioma. The expression of miR-107 has been reported to suppress the migration and invasion of glioma cells via direct degradation of the mRNA of the NOTCH2 protein. This is one of the main mechanisms of its anti-invasive action because it disrupts the NOTCH2 signaling pathway [21]. Besides NOTCH2, miR-107 also silences SALL4, a stemness factor in glioma cells, which also adds to its anti-tumor activity [22].

### Suppressing metastasis via modulation of *BDNF*/PI3K/AKT signaling

Alleviated miR-107 expression has been found to be significantly associated with low prognosis and tumor progression in NSCLC [23]. Its context-dependent tumor-suppressing action in this matter underscores the context-dependency of signaling pathways.

In this setting, miR-107 inhibits cell growth and metastasis of NSCLC cells by directly regulating *BDNF* (Brain-Derived Neurotrophic Factor). The key point to note is that *BDNF* acts as an upstream modulator of the pro-survival PI3K/AKT pathway in NSCLC. Thus, miR-107 indirectly suppresses PI3K/AKT through action on *BDNF*. This is directly opposite to what it does in other cancers, where it stimulates this very pathway. This axis was confirmed by the fact that *BDNF* overexpression was able to reverse the tumor-suppressive effects of miR-107 [23]. Other studies in NSCLC show miR-107 can also promote apoptosis by targeting the oncogene TRIAP1 [24].

### Suppressor roles by targeting cofilin-1-mediated stress responses and TFR1-driven proliferation

In cancers where the role is disputed, there is evidence of the miR-107 suppressor’s activity. The miR-107 expression is suppressed in drug-resistant HCC cell lines. It was discovered that its upregulation through mimics causes cell death, enhances ROS accumulation, and, most importantly, elevates chemosensitivity. In part, this was mediated by targeting cofilin-1 [25].

This is what can be described as the suppressor side of the CRC paradox. In other CRC studies, miR-107 is downregulated. It operates as a tumor suppressor by binding Transferrin Receptor 1 (TFR1), which is a protein implicated in cell proliferation and iron uptake. MiR-107 suppresses proliferation as well as metastasis of these CRC models by acting on TFR1 [15]. These results indicate that the functional role of miR-107 in CRC can hinge on tumor subtype or stage-dependent.

## miR-107 as an oncomiR: mechanisms of tumor progression

**Fig. 2:**
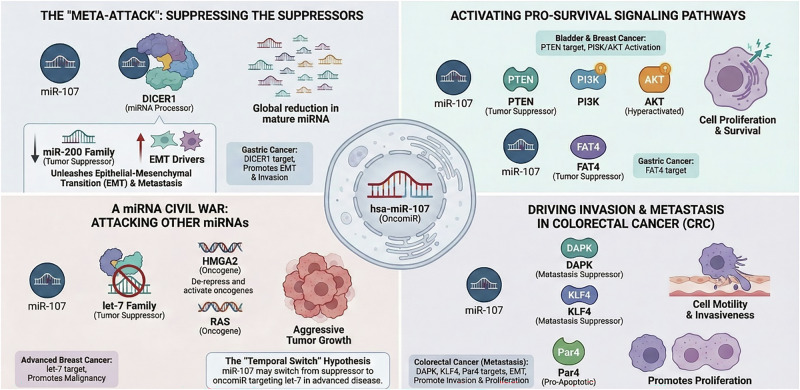
mir-107 as an oncomiR: mechanisms of tumor progression. miR-107 promotes tumor progression by suppressing miRNA biogenesis through DICER1 targeting, antagonizing tumor-suppressive miRNAs such as let-7, and activating pro-survival PI3K/AKT signaling via inhibition of PTEN and FAT4. In colorectal cancer, miR-107 further drives invasion and metastasis by repressing DAPK, KLF4, and Par4, collectively enhancing proliferation, survival, and metastatic potential.

Unlike its suppressive effect through p53, miR-107 is found in numerous aggressive malignancies as an oncomiR. In such settings, overexpression is a cause of metastasis, growth, and resistance to treatment, as shown in Fig. 2.

### A “meta-oncomiR” mechanism: the miR-103/107 family targeting of dicer

The miR-103/107 family is often found at high levels in aggressive breast, gastric, and ovarian cancers, in which it is associated with elevated metastatic capacity [6]. It is a meta-attack mechanism: the miR-103/107 family targets directly the mRNA of Dicer [26].

Dicers are the key enzyme in the final maturation of nearly all miRNAs. The miR-107 silences the biogenesis pathway in the cell by silencing Dicer expression, which causes most of the other mature miRNAs to be downregulated globally. The miR-200 family, the master suppressors of Epithelial-Mesenchymal Transition (EMT), is also widely suppressed. The miR-107 suppresses Dicer, eliminating the miR-200 family, which releases ZEB1/ZEB2 and other EMT drivers, triggering a highly metastatic phenotype [27]. This miR-107/*DICER1* axis is a particular axis that has been found to promote invasion and metastasis in gastric cancer [26].

### Activation of pro-survival signaling: the *PTEN*/PI3K/AKT pathway

The miR-103/107 are disclosed as strong oncogenes in bladder cancer. In this case, miR-107 is expressed, and it directly interacts with the 3′-UTR of the tumor suppressor *PTEN*. The main phosphatase that serves as the brake to the PI3K/AKT signaling pathway is *PTEN*. miR-107 bypasses *PTEN* and inhibits it, resulting in the hyperactivation of PI3K/AKT signaling. This facilitates the tumorigenicity, cell proliferation, and cell cycle advancement [13]. The same oncogenic pathway via *PTEN* upregulation by miR-107 that consequently phosphorylates PI3K/AKT has also been observed in breast cancer [28]. An identical result is verified in gastric cancer, where miR-107 can target another tumor suppressor, FAT4, to activate the same PI3K/AKT pathway [29].

### Activation of tumor invasiveness and metastasis: the miR-103/107/DAPK/KLF4 pathway

On the oncogenic side of the CRC conflict, numerous studies have pointed to miR-103/107 as being so-called metastamiRs, which are overexpressed in late-stage disease [14].

MiR-103/107 in this case facilitates the metastasis process by co-inhibiting two important metastasis inhibitors: DAPK (Death-Associated Protein Kinase) and KLF4 (Krueppel-like factor 4). Concomitant repression of DAPK and KLF4 has been found to instill motility and invasiveness to enable CRC metastasis in in vivo orthotopic models [14].

This mechanism shows strong clinical relevance, as a combined expression profile of high miR-103/107 with low DAPK and KLF4 serves as an independent prognostic indicator associated with lymph node and distant metastasis, reduced metastasis-free survival, and poor overall survival [14]. Other reports that miR-107 plays an oncogenic role in CRC include miR-107 stimulating proliferation via Par4 (Prostate Apoptosis Response-4) [30].

### A novel miRNA-on-miRNA interaction: targeting the let-7 suppressor family

One of the most notable oncogenic mechanisms of miR-107 is its direct trans-regulatory interaction with other microRNAs. In advanced breast cancer, miR-107 is highly expressed and shows an inverse relationship with the tumor-suppressive let-7 family, acting as an endogenous antagomir rather than competing for shared mRNA targets [17].

**Fig. 3:**
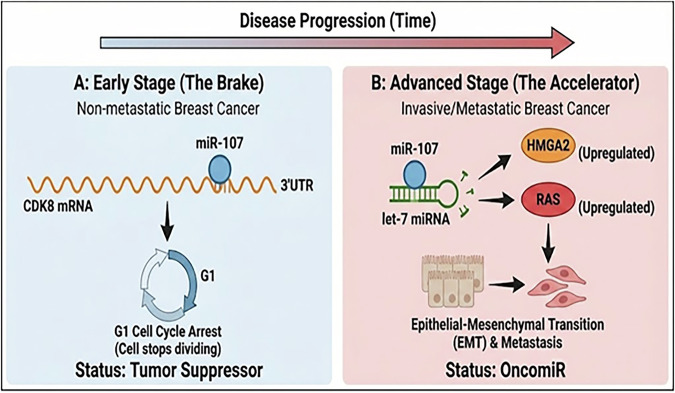
The “temporal switch” hypothesis in breast cancer progression. This schematic illustrates the dual, stage-dependent role of miR-107. **A** Early Stage: in non-metastatic contexts, miR-107 functions as a tumor suppressor by targeting the mRNA of CDK8, inducing G1 cell cycle arrest, and limiting proliferation. **B** Advanced Stage: as the tumor progresses to an aggressive phenotype, the role of miR-107 switches. It acts as an endogenous “antagomir” that directly binds and degrades the let-7 microRNA family. The loss of let-7 leads to the derepression of oncogenes such as HMGA2 and RAS, thereby driving EMT and metastasis.

This mechanism involves a direct physical interaction between mature miR-107 and mature let-7 miRNA. Binding of miR-107 to let-7, which depends on the internal loop of the miR-107/let-7 duplex, induces destabilization and degradation of let-7. As a result, miR-107 promotes derepression and upregulation of let-7 oncogenic targets, including HMGA2 and RAS, by eliminating let-7–mediated suppression. This direct inhibition of let-7 has been validated in vivo, where miR-107 expression enhances tumorigenic and metastatic potential in breast cancer mouse models [17]. Such a temporal switch—from *CDK8* suppression in early disease to let-7 inhibition in advanced stages—may explain the conflicting roles attributed to miR-107 in breast cancer (Fig. 3) [16].

## Higher-order regulation: the lncRNA “sponge” network (ceRNA)

The function of miR-107 is not just determined by its own expression, but by its availability. A crucial, higher-order regulatory network has been revealed, known as the competing endogenous RNA (ceRNA) hypothesis, where long non-coding RNAs (lncRNAs) function as “sponges” or “decoys” to sequester miRNAs [31].

This sponge mechanism delivers an influential explanation for how tumor cells can attain a functional downregulation of miR-107, even if its transcription levels are normal. By upregulating an lncRNA that binds miR-107, the cell confines it, preventing it from binding to its true mRNA targets, thereby de-repressing their expression and promoting an oncogenic phenotype [31].

### Validated lncRNA-miR-107 axes

Recent research has identified several clinically relevant lncRNA-miR-107 axes, as shown in Table 2 and Fig. 3. It was reported that the lncRNA LINC00662 is highly expressed in melanoma and acts as a molecular sponge for miR-107, leading to activation of β-catenin signaling and promoting tumor progression [32]. This sequestration “releases” miR-107’s target, the transcription factor *POU3F2*, from suppression. The resulting upregulation of POU3F2, in turn, activates the beta-catenin signaling pathway, a key driver of melanoma progression [32].

**Table 2:** The miR-107 ceRNA network: lncRNA sponges and downstream consequences.

lncRNA (sponge) vs miR-107	Cancer type	De-repressed mRNA target	Activated oncogenic pathway	Source(s)
LINC00662	Melanoma	POU3F2	β-catenin pathway	[32]
UASR1	Colorectal cancer	*CDK8*	Cell cycle progression	[33]
LINC00960	Colorectal cancer	*CDK6*	Cell cycle progression	[35]
H19	HCC	*CDK6*	Cell cycle progression	[34]
*circHIPK3*	NSCLC	BDNF	Cell proliferation and migration	[36]
MFI2-AS1	Lung cancer	NFAT5	Proliferation, angiogenesis	[37]

Additionally, the UASR1/*CDK8* axis provides a potential explanation for the CRC paradox. In CRC, the lncRNA UASR1 is regulated. UASR1 acts as a sponge for miR-107. This prevents miR-107 from binding to its tumor-suppressive target, *CDK8*. The result is a functional loss of miR-107 activity, leading to high *CDK8* expression and increased proliferation [33]. This is a sophisticated mechanism by which a cancer cell can bypass the suppressor function of miR-107.

**Fig. 4:**
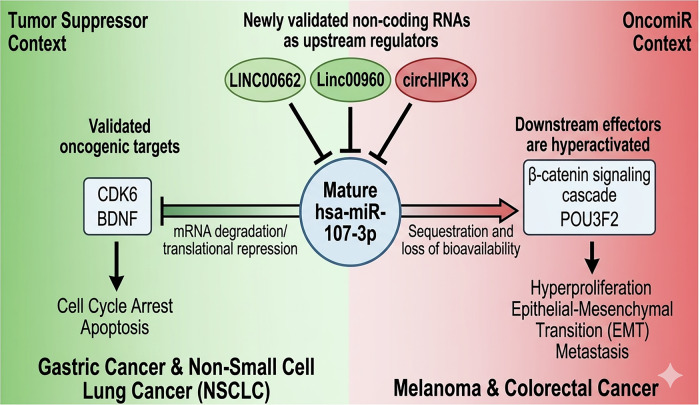
The context-dependent “switch” (the central paradox). The function of miR-107 is determined by the cellular landscape. In “suppressor contexts” (e.g., Glioma, NSCLC), miR-107 targets oncogenes such as CDK6 and BDNF, promoting cell cycle arrest and apoptosis. In “oncomiR contexts” (e.g., Bladder Cancer, Advanced Breast Cancer), miR-107 targets tumor suppressors like PTEN and DICER1. The inhibition of PTEN leads to the hyperactivation of the PI3K/AKT survival pathway, while DICER1 suppression causes global miRNA dysregulation, driving metastasis. The ceRNA network (lncRNAs like LINC00662 and UASR1) acts as an upstream regulator, “sponging” miR-107 to prevent its binding to target mRNAs.

Moreover, the H19 / *CDK6* Axis in HCC; the oncogenic lncRNA *H19* promotes proliferation in HCC by acting as a ceRNA for miR-107. By sponging miR-107, H19 de-represses the expression of its target, *CDK6*, driving cell cycle progression (Fig. 4) [34].

Recent evidence also helps to emphasize the vitality of this ceRNA process in CRC, where, by upregulating the new lncRNA *Linc00960*, miR-107 is sequestered; thus, miR-107 does not bind its target, and aggressive tumor cells continue to proliferate [35].

Also, the circular RNA *circHIPK3* is a stable molecular sponge that sequesters miR-107 in NSCLC. This sequestration results in a decrease of miR-107 bioavailability, therefore, de-repressing downstream oncogenic targets and promoting the development of lung tumors [36].

Finally, the MFI2-AS1/NFAT5 axis in lung cancer; the lncRNA MFI2-AS1 sponges miR-107, which leads to the upregulation of NFAT5, a factor that triggers proliferation and angiogenesis [37]. These complex, three-part interactions represent a new frontier in understanding miR-107 network regulation and offer novel therapeutic targets.

## Translational and clinical significance of the miR-107 network

The complex biology of miR-107 has important translational implications, positioning it as a promising clinical biomarker and a challenging yet valuable therapeutic target.

### miR-107 as a clinical biomarker

The context-specific expression of miR-107 is a potential biomarker to use in diagnosis, prognosis, and patient stratification.

#### Diagnostic value in liquid biopsies

Perhaps the most clinically immediate application for miR-107 is its use as a non-invasive biomarker in liquid biopsies. Recent data have highlighted its potential in prostate cancer (PCa). Progression of PCa to castration-resistant prostate cancer (CRPC) represents a fatal disease transition. Liquid biopsy studies have demonstrated significantly elevated circulating miR-107 levels in PCa patients compared with healthy controls. Notably, miR-107 expression is further increased in patients with advanced-stage (IV) disease and is markedly higher in CRPC compared with non-castration-resistant PCa. These findings suggest that non-invasive assessment of circulating miR-107 may serve as a promising diagnostic and prognostic biomarker for identifying and monitoring patients at risk of developing the aggressive androgen-resistant CRPC phenotype [38].

#### Prognostic value in tissue biopsies

In most of the cancers where miR-107 is an oncomiR, its elevated expression is always associated with low patient survival. Overexpression of miR-107 in Gastric Cancer is strongly associated with more metastasis and is associated with poorer disease-free survival [26]. In CRC, high miR-107 expression is also found to be an independent variable on the prognosis of CRC patients with a hazard ratio of 5.165. Its elevated concentrations have a close relationship with the pathogenesis, progression, and metastasis [39]. In addition, penile squamous cell carcinoma is characterized by elevated miR-107 expression, which correlates with higher histological grade, advanced disease stage, and reduced disease-free survival. Mechanistically, increased miR-107 levels are associated with loss of *PTEN* expression, implicating activation of the *PTEN*/PI3K/AKT oncogenic signaling pathway [40].

### A new therapeutic avenue: reversing multidrug resistance (MDR)

The miR-107 expression level has a strong correlation with the chemotherapeutic sensitivity [11]. In contexts where miR-107 functions as a tumor suppressor, its downregulation promotes drug resistance, whereas restoration of miR-107 expression re-sensitizes cancer cells to treatment. Consistently, in HCC, reduced miR-107 expression confers chemoresistance, while miR-107 upregulation enhances chemosensitivity [25]. Also in breast cancer, miR-107 mimics have been shown to augment Taxol (paclitaxel)-induced apoptosis, in part by targeting TRIAP1 and TPD52 [38].

Recent studies in gastric cancer have identified miR-107 as a potent chemosensitizer capable of overcoming MDR. MDR in gastric cancer is driven by a GATA2–CGA–EGFR positive feedback loop that sustains ERK/AKT signaling. miR-107 disrupts this loop by directly targeting GATA2 and CGA, leading to suppression of EGFR signaling and restoration of chemosensitivity to agents such as fluorouracil and Adriamycin [41, 42].

### Therapeutic development: from preclinical models to clinical challenges

Although the sophisticated biology of miR-107 can be used as a paradigm to understand regulatory functions of microRNA under different settings, the larger picture of small RNA-based therapies demonstrates enormous clinical potential in the context of targeting non-coding RNA networks in various malignancies, including oral squamous cell carcinoma (OSCC).

These miRNAs, such as oncomiRs, miR-21, and miR-34a, and tumor suppressors, miR-34a, are also associated with clinical stage and patient survival, which makes them essential diagnostic and prognostic biomarkers in OSCC [43]. Also, miR-34a-3p mimics to inhibit tumor necrosis factor-alpha (TNF-a) have consistently been shown to be effective in halting tumor progression by using therapeutic interventions to restore depleted tumor-suppressive miRNAs [44].

Moreover, these networks are therapeutically modulated in a highly complex manner by ceRNAs; an example is the upregulation of lncRNAs like OIP5-AS1 in advanced OSCC that traps tumor-suppressing miRNAs; accordingly, these multi-layered regulatory environments have to be thoroughly targeted to effectively translate the concept of small RNA therapeutics into clinical practice [45].

The promise of miR-107 as a therapeutic agent (a miR-mimic) or a therapeutic target (an anti-miR) is high, but progress is checked by the enormous complexity of miRNA-based drug delivery.

#### Preclinical efficacy of miR-107 mimics and prodrugs

In vivo preclinical studies have been promising. The subcutaneous injection of miR-107 mimics (formulated with atelocollagen) into SCID mice having subcutaneous pancreatic cancer xenografts showed a significant inhibitory effect on tumor growth relative to the controls [46].

Building on the MDR findings in gastric cancer, a follow-up in vivo study evaluated a novel miR-107 prodrug. Intratumoral administration of the miR-107 prodrug in xenograft models of multidrug-resistant gastric cancer, combined with standard chemotherapeutic agents (fluorouracil or adriamycin), resulted in significant tumor regression. This approach enhanced chemosensitivity and suppressed tumor proliferation without inducing body weight loss or detectable hepatic or renal toxicity in treated mice [42].

#### Clinical reality: the “trials and tribulations” of miRNA therapeutics

Although these findings are promising at the preclinical level, translation to clinical application remains challenging. To our present knowledge, no ongoing clinical trials of miR-107 mimics or inhibitors are present. The whole area of miRNA therapeutics has been hit by major setbacks, whereby delivery, stability, off-target, and immunotoxicity have been major challenges [1].

The warning sign to the whole profession is the destiny of MRX34, a liposome-based mimic of the tumor suppressor miR-34a. The first-in-class miRNA mimic to be introduced into human clinical trials in advanced solid tumors was MRX34 [47]. Although this Phase I trial was encouraging in terms of activity, it was discontinued in 2016 because of serious immune-mediated adverse events, which unfortunately resulted in the death of four participants. The fledgling miRNA Therapeutics stopped its activities [48].

The failure of this leaves a long shadow, implying that systemic delivery of liposomal miRNA mimics can present an intolerable risk of severe immunotoxicity. This background, however, renders the miR-107 prodrug study more important [42]. This could be because of its intratumoral delivery, which avoids systemic toxicities that dogged the MRX34 trial. This is an indication that a more conservative, local-delivery-based mode of action can be the sole way that mimic-based therapies can be proceeded with.

## Conclusion and future directions

The hsa-miR-107 role in human cancer is typified by a deep, context-specific duality; it is not a mere oncogene or tumor suppressor but a dynamic, pleiotropic component of the cell regulatory system. This review concludes that its role is an emergent property that is determined by the interaction of transcriptional input, the landscape of tissue-specific targets, and the ceRNA network. The baseline expression of p53 in a stress-response or suppressor context is determined by the status of master transcription factors, especially p53. Moreover, the mechanism of a particular cell type is determined by the presence of the transcriptome in it; miR-107 can inhibit the PI3K/AKT pathway by deactivating *BDNF* in NSCLC, but can activate the same pathway by deactivating *PTEN* in bladder cancer. Also, its functional accessibility is tightly regulated by the lncRNA environment of the so-called sponge, where the stimulation of lncRNAs such as LINC00662 or UASR1 can successfully jail miR-107 and inhibit its tumor-inhibitory effects.

**Fig. 5:**
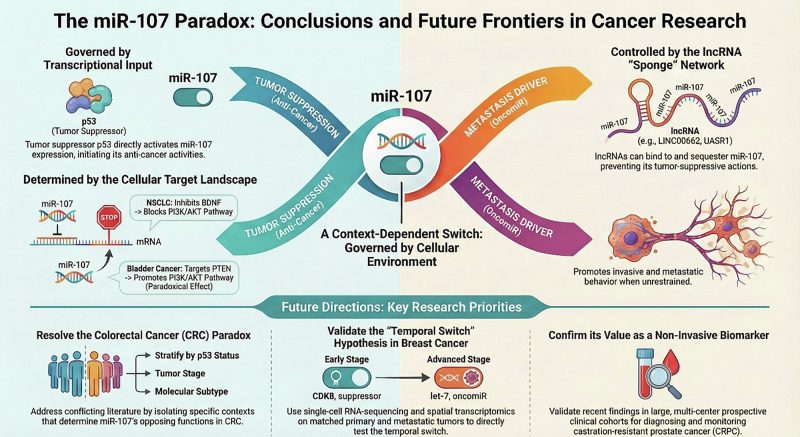
Conclusion and future directions for miR-107 in cancer research. The upper schematic illustrates miR-107 functioning as a context-dependent environmental switch. Its anti-cancer tumor-suppressive behavior is directly activated by the upstream tumor suppressor p53. Downstream, its functional output depends on the cellular target landscape: it suppresses the PI3K/Akt pathway by inhibiting BDNF in non-small cell lung cancer (NSCLC), but paradoxically activates the same pathway by targeting PTEN in bladder cancer. Conversely, its availability is restricted by the lncRNA sponge network, where molecules like LINC00662 or UASR1sequester miR-107, neutralizing its anti-cancer properties and allowing unrestrained signals to drive metastatic behavior (OncomiR). The lower panels outline three key research priorities defining future frontiers in the field: (1) resolving the colorectal cancer (CRC) paradox by stratifying patient cohorts based on p53 status, tumor stage, and molecular subtype; (2) validating the temporal switch hypothesis in breast cancer using single-cell RNA sequencing and spatial transcriptomics on matched primary and metastatic tumors to track progression from an early-stage CDK8 suppressor to an advanced let-7 oncomiR; and (3) confirming the clinical value of circulating miR-107 as a non-invasive biomarker for castration-resistant prostate cancer (CRPC) across large, multi-center prospective cohorts.

This complicated biology points to significant gaps in knowledge that create clear guidelines for future studies. To begin with, the direct contradiction in the literature on the role of miR-107 in CRC needs to be addressed by stratification of patient cohorts based on p53 mutation status, tumor stage, and molecular subtype to isolate the contexts that mediate the opposite functions of miR-107. In addition, the interesting hypothesis that miR-107 switches with time, switching between suppressors in early-stage disease targeting *CDK8* and oncomiR in advanced disease targeting *let-7*, needs to be directly verified with technologies like single-cell RNA-seq and spatial transcriptomics on matched primary and metastatic tumor samples. Lastly, the recent data that circulating miR-107 is a non-invasive CRPC biomarker is a high-impact study that now needs to be confirmed in large, multi-center, and prospective clinical cohorts to establish its diagnostic and prognostic value, as shown in Fig. 5.

## Data Availability

Data are available upon reasonable request from the corresponding author.
